# Supplementary material for: Age-related changes in the hematopoietic stem cell pool revealed via quantifying the balance of symmetric and asymmetric divisions
Source: PLoS One. 2024 Jan 29;19(1):e0292575. doi: 10.1371/journal.pone.0292575 (PMC10824414; doi:10.1371/journal.pone.0292575)
Supplement: S1 Table — The estimated values of shape parameters, αt and βt, for each age. (PDF) [file pone.0292575.s001.pdf]

Extended Data Table 1. The parameter values of beta distribution estimated by the likelihood estimation from the proportion of $CD201^{+}CD150^{+}KSL$ population.

Age (weeks)	Shape parameter 1 (α_t)	Shape parameter 2 (β_t)
4	5.42	0.620
5	0.599	0.136
6	0.304	0.175
7	0.261	0.224
8	0.497	0.204
9	0.306	0.209
15	0.387	0.354
20	0.745	0.563
22	0.862	0.152
48	0.238	0.175
52	0.169	0.164
