# Supplementary material for: Age-related changes in the hematopoietic stem cell pool revealed via quantifying the balance of symmetric and asymmetric divisions
Source: PLoS One. 2024 Jan 29;19(1):e0292575. doi: 10.1371/journal.pone.0292575 (PMC10824414; doi:10.1371/journal.pone.0292575)
Supplement: S2 Table — Based on the estimated shape parameters, α_t and β_t, for each age (shown in S1 Table), the above parameters in Eqs (3 and 4) were estimated by a nonlinear least squares method (S1 Fig). The estimated parameter values of Eqs (3 and 4) are shown. (PDF) [file pone.0292575.s002.pdf]

S2 Table. The estimated parameter values of Eqs. 3 and 4.

Parameter symbol	Value
$a_{1,\alpha}$	3.60
$a_{2,\alpha}$	0.0123
$b_{1,\alpha}$	4.98
$b_{2,\alpha}$	0.452
$a_{1,\beta}$	16.9
$a_{2,\beta}$	0.00285
$b_{1,\beta}$	0.397
$b_{2,\beta}$	0.223
