# Supplementary material for: Age-related changes in the hematopoietic stem cell pool revealed via quantifying the balance of symmetric and asymmetric divisions
Source: PLoS One. 2024 Jan 29;19(1):e0292575. doi: 10.1371/journal.pone.0292575 (PMC10824414; doi:10.1371/journal.pone.0292575)
Supplement: S3 Table — (A) Antibodies used to collect HSCs FACS. (B) Antibodies used for the analysis of 14-day culture of HSC. (C) Antibodies used for the analysis of age-related murine HSC number. (PDF) [file pone.0292575.s003.pdf]

S3 Table. List of antibodies

Table of anti-mouse antibodies used in this study including Supplier and Identifier.

a.			
Antibody	Color	Source	Indentifier
CD4	Biotin	eBioscience	Cat# 13-0041-85
CD8	Biotin	eBioscience	Cat# 13-0081-86
CD45RA/B220	Biotin	eBioscience	Cat# 36-0452-85
TER-119	Biotin	eBioscience	Cat# 13-5921-85
Ly-6G/Ly-6C (RB6-8C5)	Biotin	eBioscience	Cat# 13-5931-85
CD 127 (A7R34)	Biotin	eBioscience	Cat# 13-1271-85
CD34 (RAM34)	FITC	eBioscience	Cat#11-0341-85
Ly-6A/E (Sca-1) (D7)	PE	BioLegend	Cat#122508
c-Kit(2B8)	APC	Biolegend	Cat#105812
Streptavidin	APC/ePluor 780	eBioscience	Cat#47-4317-82
CD150 (TC15-12F12.2)	PE-Cyanine7	BioLegend	Cat#115914

b.			
Ly-6G/Ly-6C (RB6-8C5)	FITC	eBioscience	Cat#11-5931-85
TER-119	FITC	eBioscience	Cat#11-5921-82
CD4	FITC	BioLegend	Cat#100405
CD8a	FITC	BioLegend	Cat#100705
CD45R (B220)	FITC	BioLegend	Cat#103205
CD127 (A7R34)	FITC	eBioscience	Cat#11-1271-82
c-Kit(2B8)	APC	BioLegend	Cat#105812
Ly-6A/E (Sca-1) (D7)	APC-Cyanine7	BioLegend	Cat#108126
CD150 (TC15-12F12.2)	PE-Cyanine7	BioLegend	Cat#115914
CD201 (EPCR) (eBio1560 (1560))	PE	eBioscience	Cat#12-2012-82
CD11b (M1/70)	Pacific Blue	BioLegend	Cat#101224

c.			
c-Kit(2B8)	APC	BioLegend	Cat#105812
Ly-6A/E (Sca-1) (D7)	PE-Cyanine7	eBioscience	Cat#25-5981-82
CD150 (mShad150)	PE	eBioscience	Cat#12-1502-82

CD34 (RAM34)	FITC	eBioscience	Cat#11-0341-85
Ly-6G/Ly-6C (RB6-8C5)	APC-Cyanine7	eBioscience	Cat#25-5931-82
CD11b (M1/70)	APC-Cyanine7	eBioscience	Cat#25-0112-82
CD4	APC-Cyanine7	eBioscience	Cat#25-0041-82
CD8a	APC-Cyanine7	eBioscience	Cat#25-0081-82
CD45R (B220)	APC-Cyanine7	eBioscience	Cat#25-0452-82
CD127 (A7R34)	APC-Cyanine7	eBioscience	Cat#25-1271-82
TER-119	APC-Cyanine7	eBioscience	Cat#25-5921-82
