# Supplementary figures and images for: Age-related changes in the hematopoietic stem cell pool revealed via quantifying the balance of symmetric and asymmetric divisions
Source: PLoS One. 2024 Jan 29;19(1):e0292575. doi: 10.1371/journal.pone.0292575 (PMC10824414; doi:10.1371/journal.pone.0292575)

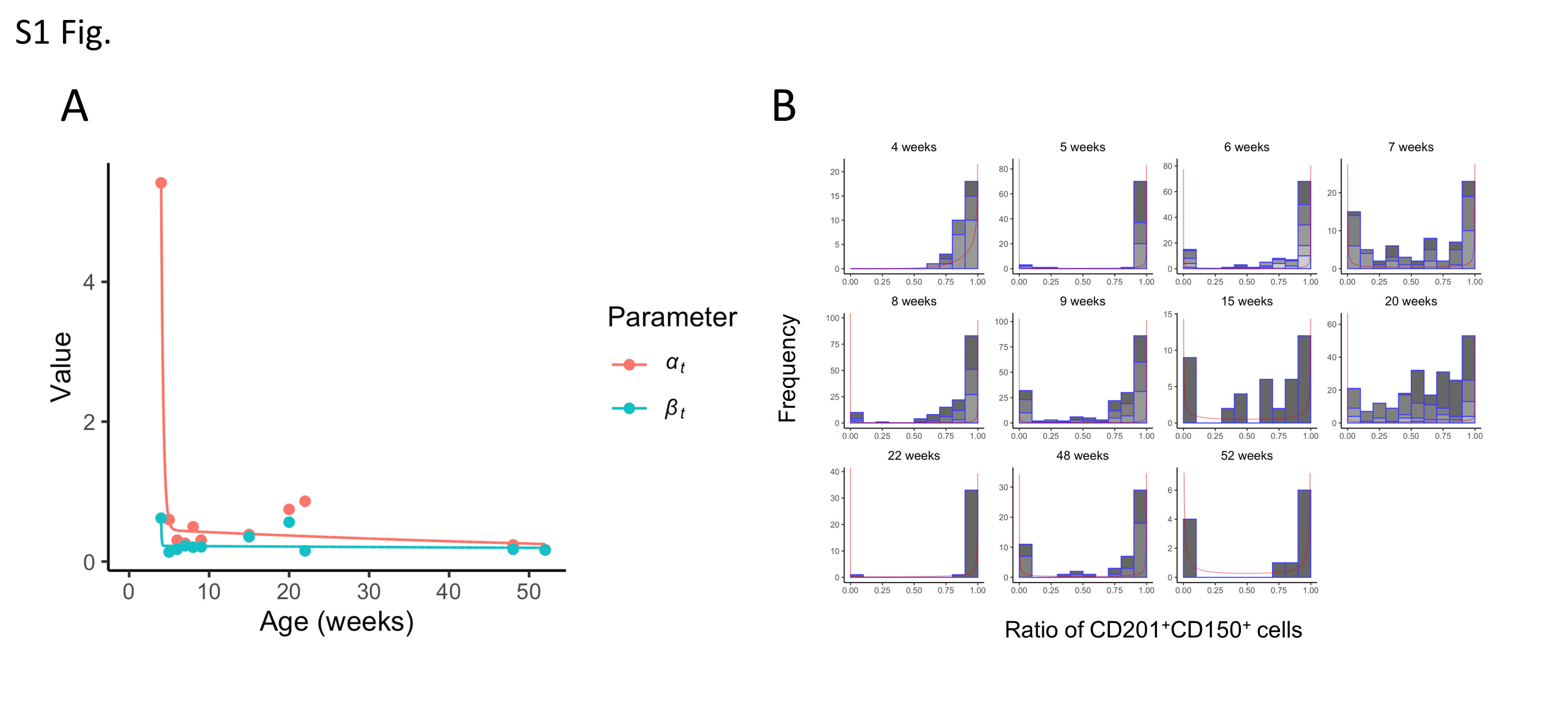

Supplement: S1 Fig — (A) The age-dependent shape parameters of beta distributions of CD201+CD150+KSL population, α(t) and β(t), were estimated. The dots and lines are corresponding to the values of shape parameters, αt and βt, estimated by maximum likelihood estimation from data with each age and Eqs (3) and (4), respectively. (B) Each figure shows the combination of the histogram of the proportion of CD201+CD150+KSL population (shown in Fig 2A) and the line derived by maximum likelihood estimation. (TIF) [file pone.0292575.s004.tif]

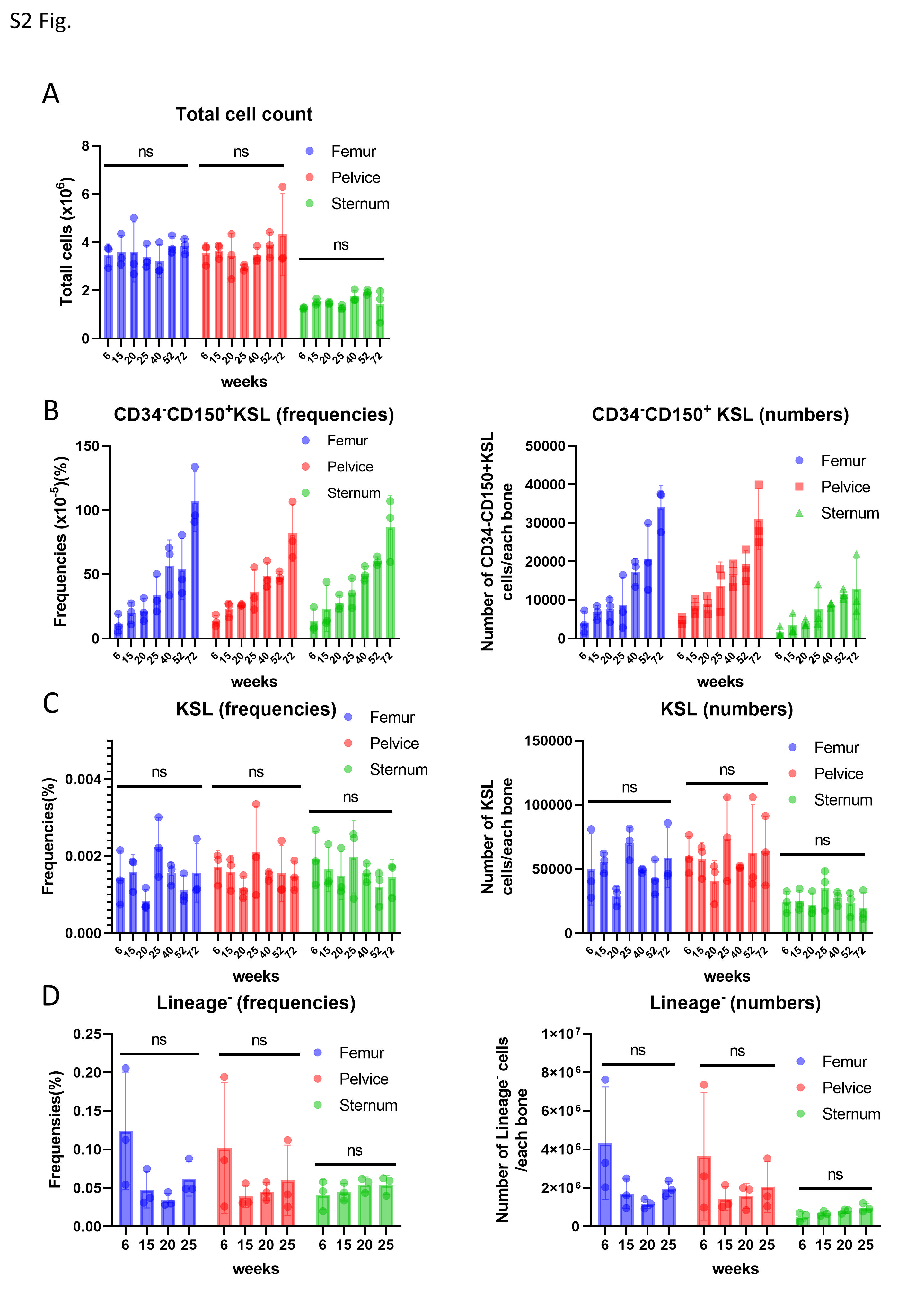

Supplement: S2 Fig — (A) The total numbers of nucleated bone marrow cells between 6 and 72 weeks of age in each bone was shown. There was no significant difference as age increased (femur, p = 0.824; pelvic, p = 0.324; and sternum, p = 0.870). The data were analyzed by one-way analysis of variance (ANOVA). If not otherwise indicated, each data point represents one independent mouse. (B) The frequencies (left) and numbers (right) of CD34-CD150+HSC between 6 and 72 weeks of age in bone marrow in each bone were shown. (C) The frequencies (left) and numbers (right) of KSL cells between 6 and 72 weeks of age in bone marrow in each bone were shown. (D) The frequencies (left) and numbers (right) of lineage-cells between 6 and 25 weeks of age in bone marrow in each bone were shown. They are similar to those of KSL cells. (TIF) [file pone.0292575.s005.tif]

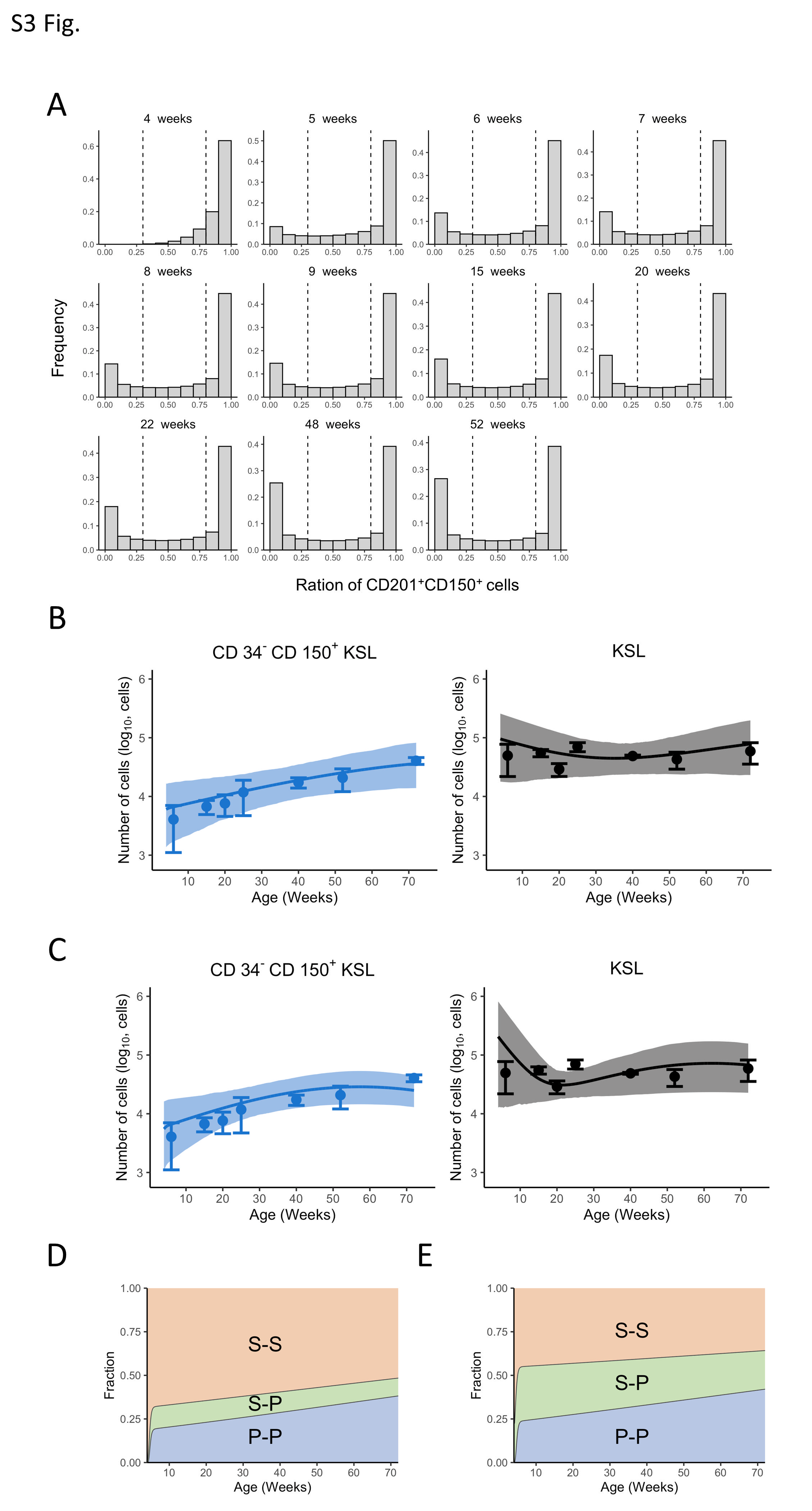

Supplement: S3 Fig — (A) The probabilities in 0.1 increments for age-dependent distributions of CD201+CD150+HSC population calculated from Eq (10) and the estimated boundaries of cell division types (i.e., the two thresholds: dashed lines) are shown. (B-E) The results of model fitting to the cell count data in the same procedure of Fig 4B and 4C with differentiation rates of progenitor cells, d, fixed as 0.05 (B, D) and 0.2 (C, E), respectively. Dots and error bars in (B) and (C) are the averages and standard deviations of the experimental data. The lines and filled area in (B) and (C) are the mean and 95% credible intervals predicted by the mathematical model. (D, E) The corresponding age-dependent distributions of S-S, S-P, and P-P divisions in mice were calculated. (TIF) [file pone.0292575.s006.tif]

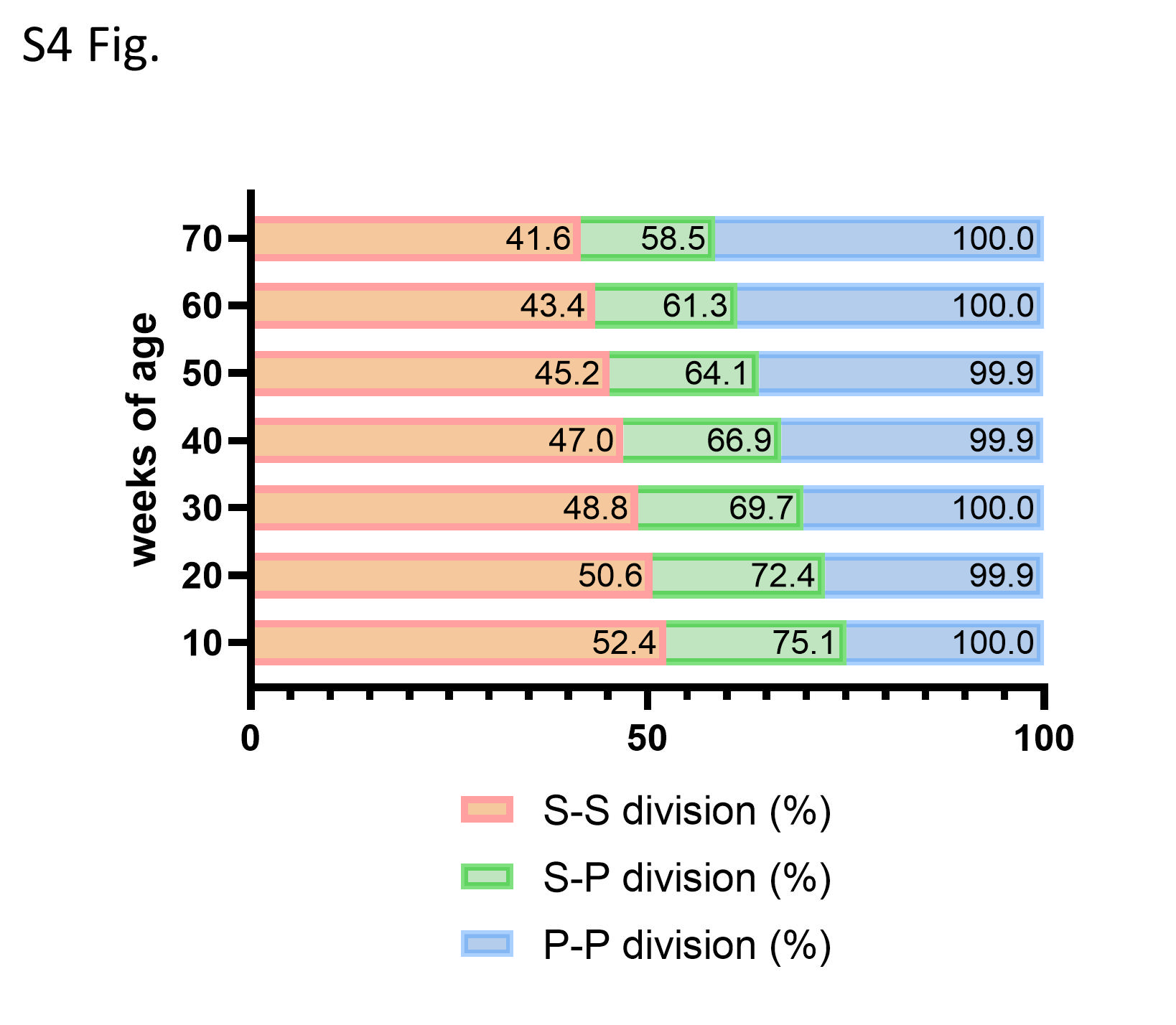

Supplement: S4 Fig — The expected distributions of S-S (red), S-P (green), and P-P (blue) divisions in mice at the specific ages 10, 20, 30, 40, 50, 60 and 70 weeks were showed from the calculation in Fig 4C. (TIF) [file pone.0292575.s007.tif]

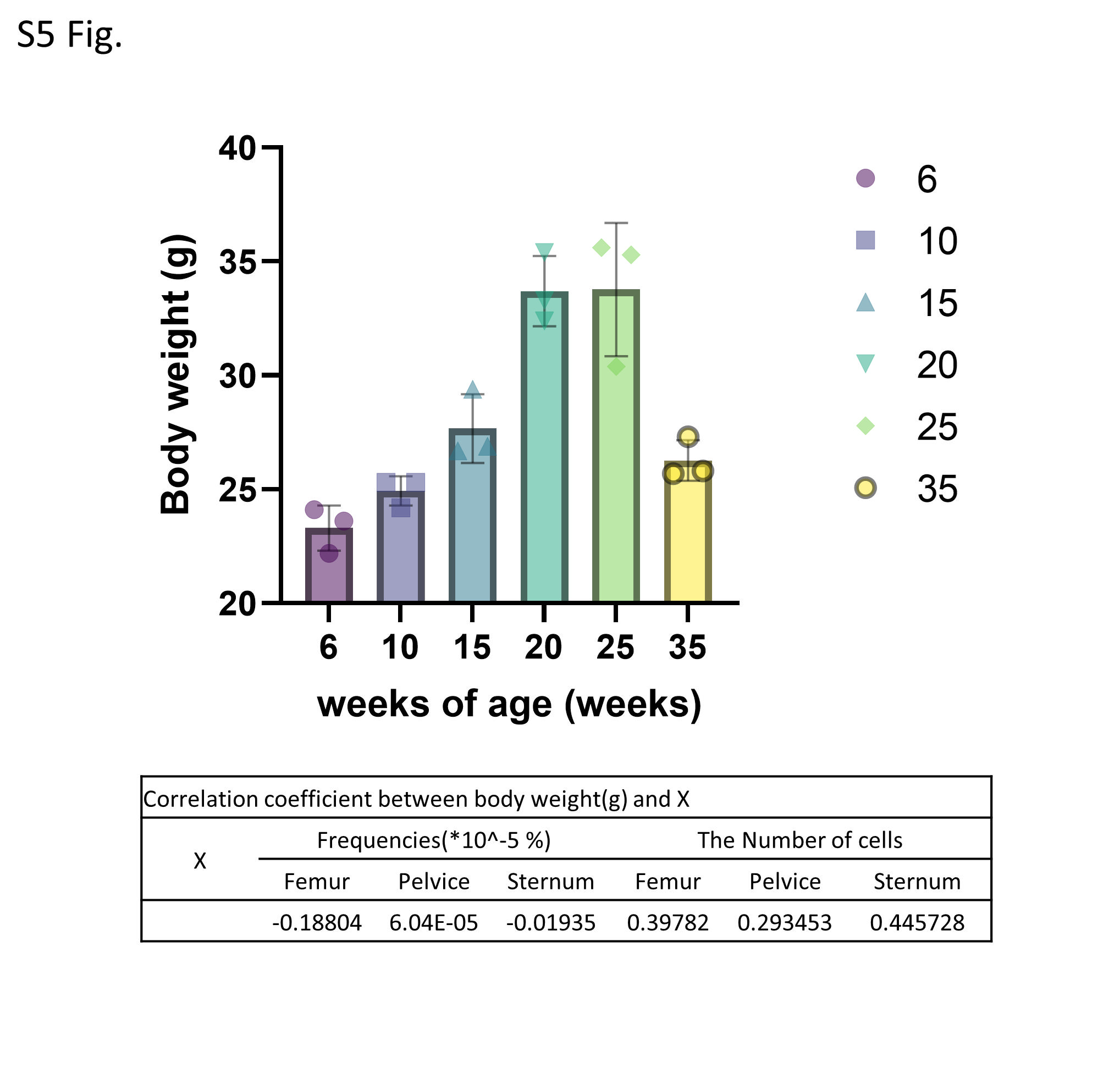

Supplement: S5 Fig — The upper panel shows the relationship between the age and body weight of mice. Three male mice were used in each age. The lower panel shows the correlation coefficient between body weight and frequencies or the number of CD34-CD150+KSL population. There is no significant correlation. (TIF) [file pone.0292575.s008.tif]
