# Supplementary material for: Age-related changes in the hematopoietic stem cell pool revealed via quantifying the balance of symmetric and asymmetric divisions
Source: PLoS One. 2024 Jan 29;19(1):e0292575. doi: 10.1371/journal.pone.0292575 (PMC10824414; doi:10.1371/journal.pone.0292575)
Supplement: S3 Data — (PDF) [file pone.0292575.s011.pdf]

weeks	S-S divisio	S-P divisio	P-P division (%)
10	52.4	22.7	24.9
20	50.6	21.8	27.5
30	48.8	20.9	30.3
40	47	19.9	33
50	45.2	18.9	35.8
60	43.4	17.9	38.7
70	41.6	16.9	41.5
