# Supplementary material for: Age-related changes in the hematopoietic stem cell pool revealed via quantifying the balance of symmetric and asymmetric divisions
Source: PLoS One. 2024 Jan 29;19(1):e0292575. doi: 10.1371/journal.pone.0292575 (PMC10824414; doi:10.1371/journal.pone.0292575)
Supplement: Attachment — Submitted filename: Kawahigashi et al., response TK.docx [file pone.0292575.s013.docx]

**Response to Reviewers**

*We would like to take the opportunity to thank all our Reviewers for their constructive comments on our manuscript.*

1 – Results, lines 146/147 : the statement is associated with figure 4A but probably should be associated with figure 4B.

As you pointed out, it was not an appropriate citation. I have revised the citation from 4A to 4B. (a line 335)

2 – Results, line 148 : description of the different parameters could be relied to Table 1.

I have added the table citation (Table 1). (a line 337)

3 – Results, line 149 : the expected HSC division distribution in presented in figure 4C, but is associated with figure 4B in the text.

I have revised the citation from 4B to 4C. (a line 337)

4 – Discussion, line 198 : reference n°41 is not related to the statement line 196-197.

You are correct. I have removed the citation No.41. (a line 397)

5 – Materials and methods, lines 270-276 : several chemicals are proposed to be tested in culture while text and figure1A show the selective use for PVA during the experiments.

I have used only PVA in the experiments. I have corrected the manuscript. (a line 94).

6 - Materials and methods, lines 285-289 : antibodies used for HSC caracterization and their manufacturer’s origin could be detailed.

I have added a list of antibodies I used as a supplemental file (lines 90, 106-108, 115-117, S3 Table).

7 - Materials and methods, line 294 : the name of the post-hoc test is not specified.

Post-hoc analysis was not done in this experiment.

8 – Figure 1B : upper pannel n°7 (from the right side) correspond to middle proportion of CD201+ CD150+ KSL, but is classified in high proportion of CD201+ CD150+ KSL

I have revised the figure.

9 - Figure 1B : contrary to the figure 1B legend, no pannel for low CD201+ CD150+ KSL ratio (purple) is proposed.

I have revised the figure 1 legend. (lines 240-241)

10 – Figure 2A : I don’t understand why the sum of all frequencies for a graph is not closed to 100. Is the ordinate value represents number of wells instead of frequency ?

Yes, as you say, the ordinate value represents number of wells. I have revised the text of the figure.

11 – Figure 2A : in the graph corresponding to mice aged of 6 weeks, the last bar (ratio 0,9-1) shows 5 parts while it is given for 4 mice in the legend. Is there 5 mice for this age?

The legend was wrong. I used 5 mice for the experiment. I have revised the legend. (line 256)

12- Figure 3B and C : maybe the authors could precise in the legend that the right pannel represent the same data compared to left pannel, with log10 transformation of data.

I have revised the legend (Figure 3B and C) as you suggested. (lines 295-296 and 298-299)

13 – Figure S1B : there is no description in the legend for this part.

I have added the description of S1B. (lines 611-615)

14 – Figure S5 : there is no legend provided for this figure, and it is not quoted in the text.

I have added the explanation about Fig S5 and a legend. It was inserted to show that there is not significant correlation between body weight and the number of HSC of mice. (lines 286-287, 641-644)
